# Supplementary material for: Genome-wide analyses of LATERAL ORGAN BOUNDARIES in cassava reveal the role of LBD47 in defence against bacterial blight
Source: PLoS One. 2023 Apr 20;18(4):e0282100. doi: 10.1371/journal.pone.0282100 (PMC10118128; doi:10.1371/journal.pone.0282100)
Supplement: S1 File — (DOCX) [file pone.0282100.s001.docx]

# Supplementary Materials

## Table S1. Functional characterisation of several *LBD* members in different species

| **Genes** | **Species** | **Functions** | **References** |
| --- | --- | --- | --- |
| LOB (ASL4) | Arabidopsis | Leaf development | (1) |
| LBD3 (ASL9) | Arabidopsis | Flower development, cytokinin response | (2) |
| LBD6 (ASL2) | Arabidopsis | Leaf formation | (3, 4) |
| LBD12 (ASL5) | Arabidopsis | Leaf formation | (5) |
| LBD16 (ASL18) | Arabidopsis | Root formation, auxin response | (6, 7) |
| LBD18 (ASL20) | Arabidopsis | Root formation, auxin response | (7, 8) |
| LBD30 (JLO/ASL19) | Arabidopsis | Embryogenesis and floral development | (9, 10) |
| LBD29 (ASL16) | Arabidopsis | Root formation, auxin response | (6, 7) |
| LBD36 (ASL1) | Arabidopsis | Flower development | (6) |
| LBD37 (ASL39) | Arabidopsis | Metabolism | (11) |
| LBD38 (ASL40) | Arabidopsis | Metabolism | (11) |
| LBD39 (ASL41) | Arabidopsis | Metabolism | (11) |
| LBD40 (ASL37) | Arabidopsis | Gibberellin response | (12) |
| LBD10, LBD27 | Arabidopsis | Pollen development | (13, 14) |
| LBD16, LBD17, LBD18, LBD29 | Arabidopsis | Plant regeneration | (15, 16) |
| LBD20, LBD16 | Arabidopsis | Disease susceptibility | (17, 18) |
| LBD25 | Arabidopsis | Photomorphogenesis | (19) |
| LOB1 | Citrus | Disease susceptibility | (20) |
| Rtcs | Maize | Shoot-borne and seminal root initiation | (21) |
| Ig1 | Maize | Flower development | (22) |
| Ra2 | Maize | Flower development | (23) |
| ARL1 (CRL1) | Rice | Root formation | (24, 25) |
| DH1 | Rice | Flower development | (26) |
| LBD37 | Rice | Metabolism | (27) |
| AS2 | Rice | Leaf development | (28) |
| ELP1/PLP | Medicago | Pulvinus identity | (29, 30) |
| ELP1/PLP | Medicago | Petiole development | (31) |
| APU | Pea | Pulvinus identity | (29) |
| SLP | Lotus | Pulvinus identity | (29) |
| LBD50 | Salvia | Metabolism | (32) |
| LBD123 | Cotton | Metabolism | (33) |

1. Shuai B, Reynaga-Pena CG, Springer PSJPp. The lateral organ boundaries gene defines a novel, plant-specific gene family. 2002;129(2):747-61.

2. Naito T, Yamashino T, Kiba T, Koizumi N, Kojima M, Sakakibara H, et al. A link between cytokinin and ASL9 (ASYMMETRIC LEAVES 2 LIKE 9) that belongs to the AS2/LOB (LATERAL ORGAN BOUNDARIES) family genes in Arabidopsis thaliana. 2007;71(5):1269-78.

3. Semiarti E, Ueno Y, Tsukaya H, Iwakawa H, Machida C, Machida YJD. The ASYMMETRIC LEAVES2 gene of Arabidopsis thaliana regulates formation of a symmetric lamina, establishment of venation and repression of meristem-related homeobox genes in leaves. 2001;128(10):1771-83.

4. Ori N, Eshed Y, Chuck G, Bowman JL, Hake SJD. Mechanisms that control knox gene expression in the Arabidopsis shoot. 2000;127(24):5523-32.

5. Nakazawa M, Ichikawa T, Ishikawa A, Kobayashi H, Tsuhara Y, Kawashima M, et al. Activation tagging, a novel tool to dissect the functions of a gene family. 2003;34(5):741-50.

6. Okushima Y, Fukaki H, Onoda M, Theologis A, Tasaka MJTPC. ARF7 and ARF19 regulate lateral root formation via direct activation of LBD/ASL genes in Arabidopsis. 2007;19(1):118-30.

7. Okushima Y, Overvoorde PJ, Arima K, Alonso JM, Chan A, Chang C, et al. Functional genomic analysis of the AUXIN RESPONSE FACTOR gene family members in Arabidopsis thaliana: unique and overlapping functions of ARF7 and ARF19. 2005;17(2):444-63.

8. Lee HW, Kim NY, Lee DJ, Kim JJPp. LBD18/ASL20 regulates lateral root formation in combination with LBD16/ASL18 downstream of ARF7 and ARF19 in Arabidopsis. 2009;151(3):1377-89.

9. Borghi L, Bureau M, Simon RdJTPC. Arabidopsis JAGGED LATERAL ORGANS is expressed in boundaries and coordinates KNOX and PIN activity. 2007;19(6):1795-808.

10. Soyano T, Thitamadee S, Machida Y, Chua N-HJTPC. Asymmetric leaves2-like19/lateral organ boundaries domain30 and ASL20/LBD18 regulate tracheary element differentiation in Arabidopsis. 2008;20(12):3359-73.

11. Rubin G, Tohge T, Matsuda F, Saito K, Scheible W-RdJTPC. Members of the LBD family of transcription factors repress anthocyanin synthesis and affect additional nitrogen responses in Arabidopsis. 2009;21(11):3567-84.

12. Zentella R, Zhang Z-L, Park M, Thomas SG, Endo A, Murase K, et al. Global analysis of DELLA direct targets in early gibberellin signaling in Arabidopsis. 2007;19(10):3037-57.

13. Kim MJ, Kim M, Lee MR, Park SK, Kim JJTPJ. LATERAL ORGAN BOUNDARIES DOMAIN (LBD) 10 interacts with SIDECAR POLLEN/LBD 27 to control pollen development in A rabidopsis. 2015;81(5):794-809.

14. Oh SA, Park KS, Twell D, Park SKJTPJ. The SIDECAR POLLEN gene encodes a microspore‐specific LOB/AS2 domain protein required for the correct timing and orientation of asymmetric cell division. 2010;64(5):839-50.

15. Fan M, Xu C, Xu K, Hu YJCr. LATERAL ORGAN BOUNDARIES DOMAIN transcription factors direct callus formation in Arabidopsis regeneration. 2012;22(7):1169-80.

16. Liu J, Sheng L, Xu Y, Li J, Yang Z, Huang H, et al. WOX11 and 12 are involved in the first-step cell fate transition during de novo root organogenesis in Arabidopsis. 2014;26(3):1081-93.

17. Thatcher LF, Powell JJ, Aitken EA, Kazan K, Manners JMJPp. The lateral organ boundaries domain transcription factor LBD20 functions in Fusarium wilt susceptibility and jasmonate signaling in Arabidopsis. 2012;160(1):407-18.

18. Cabrera J, Díaz‐Manzano FE, Sanchez M, Rosso MN, Melillo T, Goh T, et al. A role for LATERAL ORGAN BOUNDARIES‐DOMAIN 16 during the interaction A rabidopsis–M eloidogyne spp. provides a molecular link between lateral root and root‐knot nematode feeding site development. 2014;203(2):632-45.

19. Mangeon A, Bell EM, Lin W-c, Jablonska B, Springer PSJJoeb. Misregulation of the LOB domain gene DDA1 suggests possible functions in auxin signalling and photomorphogenesis. 2011;62(1):221-33.

20. Hu Y, Zhang J, Jia H, Sosso D, Li T, Frommer WB, et al. Lateral organ boundaries 1 is a disease susceptibility gene for citrus bacterial canker disease. 2014;111(4):E521-E9.

21. Taramino G, Sauer M, Stauffer Jr JL, Multani D, Niu X, Sakai H, et al. The maize (Zea mays L.) RTCS gene encodes a LOB domain protein that is a key regulator of embryonic seminal and post‐embryonic shoot‐borne root initiation. 2007;50(4):649-59.

22. Evans MMJTPC. The indeterminate gametophyte1 gene of maize encodes a LOB domain protein required for embryo sac and leaf development. 2007;19(1):46-62.

23. Bortiri E, Chuck G, Vollbrecht E, Rocheford T, Martienssen R, Hake SJTPC. ramosa2 encodes a LATERAL ORGAN BOUNDARY domain protein that determines the fate of stem cells in branch meristems of maize. 2006;18(3):574-85.

24. Inukai Y, Sakamoto T, Ueguchi-Tanaka M, Shibata Y, Gomi K, Umemura I, et al. Crown rootless1, which is essential for crown root formation in rice, is a target of an AUXIN RESPONSE FACTOR in auxin signaling. 2005;17(5):1387-96.

25. Liu H, Wang S, Yu X, Yu J, He X, Zhang S, et al. ARL1, a LOB‐domain protein required for adventitious root formation in rice. 2005;43(1):47-56.

26. Li A, Zhang Y, Wu X, Tang W, Wu R, Dai Z, et al. DH1, a LOB domain-like protein required for glume formation in rice. 2008;66(5):491-502.

27. Albinsky D, Kusano M, Higuchi M, Hayashi N, Kobayashi M, Fukushima A, et al. Metabolomic screening applied to rice FOX Arabidopsis lines leads to the identification of a gene-changing nitrogen metabolism. 2010;3(1):125-42.

28. Ma Y, Wang F, Guo J, Zhang XSJJoPB. Rice OsAS2 gene, a member of LOB domain family, functions in the regulation of shoot differentiation and leaf development. 2009;52(5):374-81.

29. Chen J, Moreau C, Liu Y, Kawaguchi M, Hofer J, Ellis N, et al. Conserved genetic determinant of motor organ identity in Medicago truncatula and related legumes. 2012;109(29):11723-8.

30. Zhou C, Han L, Fu C, Chai M, Zhang W, Li G, et al. Identification and characterization of petiolule‐like pulvinus mutants with abolished nyctinastic leaf movement in the model legume Medicago truncatula. 2012;196(1):92-100.

31. Ge L, Peng J, Berbel A, Madueño F, Chen RJPp. Regulation of compound leaf development by PHANTASTICA in Medicago truncatula. 2014;164(1):216-28.

32. Lu X-Y, Liang X-Y, Li X, Shen P-X, Cao X-Y, Chen C, et al. Genome-wide characterisation and expression profiling of the LBD family in Salvia miltiorrhiza reveals the function of LBD50 in jasmonate signaling and phenolic biosynthesis. 2020;144:112006.

33. Yu J, Xie Q, Li C, Dong Y, Zhu S, Chen JJP. Comprehensive characterization and gene expression patterns of LBD gene family in Gossypium. 2020;251(4):1-16.

## Table S2. Primers used for quantitive real-time PCR and Vector construction

| **Primer name** | **Primer sequence (5'-3')** |
| --- | --- |
|  |  |
| pCsCMV-PDS-F | AGTGGTCTCTGTCCAGTCCTTATGCAAGCCTGTTTCATACCAGTC |
| pCsCMV-*PDS*-R | GGTCTCAGCAGACCACAAGTACTTTGTACGGGAGTGTTTCTGCAT |
|  |  |
| pCsCMV-*LBD47*-F | AGTGGTCTCTGTCCAGTCCTGACGAAGTGTCTGAGGCTGAA |
| pCsCMV-*LBD47*-R | GGTCTCAGCAGACCACAAGTCGGTTTACGAGGCGATGA |
|  |  |
| *MeLBD1-F* | AGCCTCACAAGTTTGCCAGT |
| *MeLBD1-R* | AACTCACCGCATCACTTCGT |
|  |  |
| *MeLBD2-F* | TGCCCACGACTGTGTTTTTG |
| *MeLBD2-R* | CATTGAACTCACCGCATCGC |
|  |  |
| *MeLBD12-F* | TATGAGTCCTTGTGCGGCTT |
| *MeLBD12-R* | CGAACACGCGATGAGCAATG |
|  |  |
| *MeLBD13-F* | CAGATGCAGTAAGCAGCATGG |
| *MeLBD13-R* | GCTTGTGCCTTGGCTAGTTG |
|  |  |
| *MeLBD46-F* | GCTAAGTTCTTTGGCCGTGC |
| *MeLBD46-R* | AGCGCCGTTTACAGGATTCA |
|  |  |
| *MeLBD47-F* | AGTGGATCGAGACCCCTGAA |
| *MeLBD47-R* | GAAACAAAGCAGGACGCTGG |
|  |  |
| *GAD1-F* | AGTGGTGGGTTTATTGCCCC |
| *GAD1-R* | TGAGGGTGAAGGTAGGCTGA |
|  |  |
| *TFIIA-S-F* | TGGCGACGTTTGAGCTATACA |
| *TFIIA-S-R* | GCTGAGAGTACCGCTCTGAA |
|  |  |
| *HSFC1-F* | CGCAGCTCCTCCTCCATATT |
| *HSFC1-R* | GCCCCAGGTGATAAGTGTGT |
|  |  |
| *HSP90-F* | AAACTCGGCGCTGTTCCTAA |
| *HSP90-R* | GACACCTCAGCCTGGAACTC |

## Table S3. Transcriptome datasets used for expression analysis and WGCNA assay. Run IDs were assigned by NCBI GEO.

| **Run ID1** | **Sample name** | **Note** | **Reference** |
| --- | --- | --- | --- |
| SRR1298996 | W14_root | Expression analysis | Wang et al. (1) |
| SRR1298998 | W14_leaf |  |  |
| SRR1299000 | KU50_leaf |  |  |
| SRR1299001 | KU50_root75d |  |  |
| SRR1299002 | KU50_root120d |  |  |
| SRR1299003 | KU50_root150d |  |  |
| SRR1299005 | Arg7_root_early |  |  |
| SRR1299006 | Arg7_root_middle |  |  |
| SRR1299007 | Arg7_root_late |  |  |
| SRR1299009 | Arg7_leaf |  |  |
| SRR1537519 | mock_8hr | Expression analysis and WGCNA assay | Cohn et al. (2) |
| SRR1538456 | mock_8hr |  |  |
| SRR1538457 | mock_8hr |  |  |
| SRR1544068 | mock_8hr |  |  |
| SRR1538484 | Xam668_8hr |  |  |
| SRR1538485 | Xam668_8hr |  |  |
| SRR1538486 | Xam668_8hr |  |  |
| SRR1538487 | X. euvesicatoria_8hr |  |  |
| SRR1538488 | X. euvesicatoria_8hr |  |  |
| SRR1538489 | X. euvesicatoria_8hr |  |  |
| SRR1538490 | X. euvesicatoria(TAL20_Xam668)_8hr |  |  |
| SRR1538491 | X. euvesicatoria(TAL20_Xam668)_8hr |  |  |
| SRR1539556 | X. euvesicatoria(TAL20_Xam668)_8hr |  |  |
| SRR1538828 | mock_24hr |  |  |
| SRR1538829 | mock_24hr |  |  |
| SRR1538848 | mock_24hr |  |  |
| SRR1538903 | X. euvesicatoria_24hr |  |  |
| SRR1538904 | X. euvesicatoria_24hr |  |  |
| SRR1538905 | X. euvesicatoria_24hr |  |  |
| SRR1538928 | X. euvesicatoria(TAL20_Xam668)_24hr |  |  |
| SRR1538929 | X. euvesicatoria(TAL20_Xam668)_24hr |  |  |
| SRR1538930 | X. euvesicatoria(TAL20_Xam668)_24hr |  |  |
| SRR1538931 | Xam668_24hr |  |  |
| SRR1538932 | Xam668_24hr |  |  |
| SRR1538933 | Xam668_24hr |  |  |
| SRR1539557 | mock_50hr |  |  |
| SRR1539558 | mock_50hr |  |  |
| SRR1539559 | mock_50hr |  |  |
| SRR1539560 | Xam668_50hr |  |  |
| SRR1539561 | Xam668_50hr |  |  |
| SRR1539562 | Xam668_50hr |  |  |
| SRR1539563 | X. euvesicatoria_50hr |  |  |
| SRR1539564 | X. euvesicatoria_50hr |  |  |
| SRR1539565 | X. euvesicatoria_50hr |  |  |
| SRR1539566 | X. euvesicatoria(TAL20_Xam668)_50hr |  |  |
| SRR1539567 | X. euvesicatoria(TAL20_Xam668)_50hr |  |  |
| SRR1539568 | X. euvesicatoria(TAL20_Xam668)_50hr |  |  |

1. Wang W, Feng B, Xiao J, Xia Z, Zhou X, Li P, et al. Cassava genome from a wild ancestor to cultivated varieties. 2014;5(1):1-9.

2. Cohn M, Bart RS, Shybut M, Dahlbeck D, Gomez M, Morbitzer R, et al. Xanthomonas axonopodis virulence is promoted by a transcription activator-like effector–mediated induction of a SWEET sugar transporter in cassava. 2014;27(11):1186-98.

## Table S4. Homologous blocks containing cassava LBD genes

| **Block code** | **Homologous block A** |  |  | **Homologous block B** |  |  | **Homologous gene pair** |  | **Ka / Ks** |
| --- | --- | --- | --- | --- | --- | --- | --- | --- | --- |
|  | **Chr.** | **Start** | **End** | **Chr.** | **Start** | **End** |  |  |  |
| 1 | Chr1 | 23137090 | 23138516 | Chr14 | 8273112 | 8274407 | Manes.01G110000 | Manes.14G102500 | 0.195950314 |
| 2 | Chr1 | 23137090 | 23138516 | Chr2 | 5106630 | 5108019 | Manes.01G110000 | Manes.02G068400 | 0.15364927 |
| 3 | Chr1 | 33740451 | 33743054 | Chr5 | 3254262 | 3256710 | Manes.01G265300 | Manes.05G044500 | 0.141544862 |
| 4 | Chr1 | 23137090 | 23138516 | Chr6 | 18330826 | 18331652 | Manes.01G110000 | Manes.06G068500 | 0.189929451 |
| 5 | Chr2 | 5106630 | 5108019 | Chr6 | 18330826 | 18331652 | Manes.02G068400 | Manes.06G068500 | 0.114585729 |
| 6 | Chr3 | 4122624 | 4123494 | Chr4 | 26684413 | 26685168 | Manes.03G048400 | Manes.04G140100 | 0.287586147 |
| 7 | Chr4 | 2645577 | 2647259 | Chr7 | 23105287 | 23106645 | Manes.04G025300 | Manes.07G102200 | 0.088171172 |
| 8 | Chr8 | 31801958 | 31803707 | Chr9 | 25748784 | 25749881 | Manes.08G154100 | Manes.09G137400 | 0.131566997 |
| 9 | Chr10 | 3830274 | 3831489 | Chr11 | 25328208 | 25329869 | Manes.10G042900 | Manes.11G140800 | 0.149256351 |
| 10 | Chr10 | 3830274 | 3831489 | Chr4 | 2645577 | 2647259 | Manes.10G042900 | Manes.04G025300 | 0.099196926 |
| 11 | Chr10 | 3830274 | 3831489 | Chr7 | 23105287 | 23106645 | Manes.10G042900 | Manes.07G102200 | 0.1737791 |
| 12 | Chr10 | 441952 | 443794 | Chr7 | 26403736 | 26405477 | Manes.10G005700 | Manes.07G138500 | 0.082547991 |
| 13 | Chr10 | 680175 | 680863 | Chr7 | 25912090 | 25912774 | Manes.10G008800 | Manes.07G134700 | 0.094401032 |
| 14 | Chr10 | 794939 | 795796 | Chr7 | 25697536 | 25698858 | Manes.10G010200 | Manes.07G132200 | 0.162741666 |
| 15 | Chr10 | 2238554 | 2240468 | Chr7 | 24652426 | 24653392 | Manes.10G027400 | Manes.07G118600 | 0.359906896 |
| 16 | Chr11 | 2272404 | 2273147 | Chr16 | 23816043 | 23816684 | Manes.11G026000 | Manes.16G081100 | 0.534216986 |
| 17 | Chr11 | 2272404 | 2273147 | Chr3 | 4122624 | 4123494 | Manes.11G026000 | Manes.03G048400 | 0.353296498 |
| 18 | Chr11 | 2272404 | 2273147 | Chr4 | 26684413 | 26685168 | Manes.11G140800 | Manes.04G140100 | NaN |
| 19 | Chr11 | 25328208 | 25329869 | Chr4 | 2645577 | 2647259 | Manes.11G140800 | Manes.04G025300 | 0.070835361 |
| 20 | Chr12 | 2373012 | 2375258 | Chr13 | 2995261 | 2997536 | Manes.12G030700 | Manes.13G031900 | 0.190105638 |
| 21 | Chr12 | 25605195 | 25606435 | Chr13 | 23990699 | 23991927 | Manes.12G110600 | Manes.13G113000 | 0.149203606 |
| 22 | Chr12 | 14936620 | 14938922 | Chr13 | 25544935 | 25547012 | Manes.12G093700 | Manes.13G128100 | 0.160367377 |
| 23 | Chr12 | 13976847 | 13979498 | Chr17 | 18875432 | 18876190 | Manes.12G091800 | Manes.17G051500 | 0.467755807 |
| 24 | Chr12 | 25605195 | 25606435 | Chr5 | 24762145 | 24763119 | Manes.12G110600 | Manes.05G180800 | NaN |
| 25 | Chr13 | 23990699 | 23991927 | Chr18 | 3885935 | 3886979 | Manes.13G113000 | Manes.18G045200 | NaN |
| 26 | Chr13 | 23990699 | 23991927 | Chr5 | 24762145 | 24763119 | Manes.13G113000 | Manes.05G180800 | NaN |
| 27 | Chr14 | 8273112 | 8274407 | Chr2 | 5106630 | 5108019 | Manes.14G102500 | Manes.02G068400 | 0.143818045 |
| 28 | Chr14 | 5060059 | 5062273 | Chr6 | 21828155 | 21830418 | Manes.14G062100 | Manes.06G108700 | 0.192260297 |
| 29 | Chr14 | 5942407 | 5943402 | Chr6 | 20914462 | 20915524 | Manes.14G073600 | Manes.06G097500 | 0.103844384 |
| 30 | Chr14 | 8273112 | 8274407 | Chr6 | 18330826 | 18331652 | Manes.14G102500 | Manes.06G068500 | 0.167065548 |
| 31 | Chr14 | 1166656 | 1169919 | Chr6 | 27305151 | 27308303 | Manes.14G012200 | Manes.06G173400 | 0.081959604 |
| 32 | Chr15 | 11786336 | 11788234 | Chr17 | 25312798 | 25314680 | Manes.15G149900 | Manes.17G112700 | 0.212968941 |
| 33 | Chr16 | 23816043 | 23816684 | Chr3 | 4122624 | 4123494 | Manes.16G081100 | Manes.03G048400 | 0.415017156 |
| 34 | Chr16 | 23816043 | 23816684 | Chr4 | 26684413 | 26685168 | Manes.16G081100 | Manes.04G140100 | 0.374851554 |
| 35 | Chr18 | 3885935 | 3886979 | Chr5 | 24762145 | 24763119 | Manes.18G045200 | Manes.05G180800 | 0.156225206 |

## Table S5. List of genes used for WGCNA analysis

| **Genes Identity** |
| --- |
| Manes.S108600 |
| Manes.S078700 |
| Manes.S052900 |
| Manes.S022900 |
| Manes.S012600 |
| Manes.18G141200 |
| Manes.18G121700 |
| Manes.18G072700 |
| Manes.18G064400 |
| Manes.18G057000 |
| Manes.18G044500 |
| Manes.18G041700 |
| Manes.18G036100 |
| Manes.18G029700 |
| Manes.18G012200 |
| Manes.18G002900 |
| Manes.17G119500 |
| Manes.17G102800 |
| Manes.17G100500 |
| Manes.17G090500 |
| Manes.17G074400 |
| Manes.17G065500 |
| Manes.17G038000 |
| Manes.17G026600 |
| Manes.17G025700 |
| Manes.17G001600 |
| Manes.16G131900 |
| Manes.16G129800 |
| Manes.16G120400 |
| Manes.16G116200 |
| Manes.16G115300 |
| Manes.16G110200 |
| Manes.16G102700 |
| Manes.16G087600 |
| Manes.16G074300 |
| Manes.16G062800 |
| Manes.16G059200 |
| Manes.16G050400 |
| Manes.16G042400 |
| Manes.16G040500 |
| Manes.16G030600 |
| Manes.16G012700 |
| Manes.16G004100 |
| Manes.16G003100 |
| Manes.15G185800 |
| Manes.15G156600 |
| Manes.15G151200 |
| Manes.15G142500 |
| Manes.15G140700 |
| Manes.15G132600 |
| Manes.15G131100 |
| Manes.15G121100 |
| Manes.15G117700 |
| Manes.15G116800 |
| Manes.15G101800 |
| Manes.15G090500 |
| Manes.15G084200 |
| Manes.15G076500 |
| Manes.15G050800 |
| Manes.15G048300 |
| Manes.15G039300 |
| Manes.15G034300 |
| Manes.15G028700 |
| Manes.15G016100 |
| Manes.15G011500 |
| Manes.15G007400 |
| Manes.14G171400 |
| Manes.14G150400 |
| Manes.14G143400 |
| Manes.14G135100 |
| Manes.14G121400 |
| Manes.14G113500 |
| Manes.14G101900 |
| Manes.14G078500 |
| Manes.14G064800 |
| Manes.14G063800 |
| Manes.14G060500 |
| Manes.14G037400 |
| Manes.14G035500 |
| Manes.14G031800 |
| Manes.14G028600 |
| Manes.14G021300 |
| Manes.14G019800 |
| Manes.14G018200 |
| Manes.14G009100 |
| Manes.14G006900 |
| Manes.13G143900 |
| Manes.13G143500 |
| Manes.13G138100 |
| Manes.13G135600 |
| Manes.13G132200 |
| Manes.13G116500 |
| Manes.13G113000 |
| Manes.13G087000 |
| Manes.13G084100 |
| Manes.13G060800 |
| Manes.13G057700 |
| Manes.13G052000 |
| Manes.13G042800 |
| Manes.13G019600 |
| Manes.13G010400 |
| Manes.13G001300 |
| Manes.13G000300 |
| Manes.12G139200 |
| Manes.12G134900 |
| Manes.12G131000 |
| Manes.12G114400 |
| Manes.12G111400 |
| Manes.12G110600 |
| Manes.12G101400 |
| Manes.12G068900 |
| Manes.12G047600 |
| Manes.12G043800 |
| Manes.12G043700 |
| Manes.12G035600 |
| Manes.12G023600 |
| Manes.12G023300 |
| Manes.12G015500 |
| Manes.12G009700 |
| Manes.11G162900 |
| Manes.11G162400 |
| Manes.11G131500 |
| Manes.11G096200 |
| Manes.11G085500 |
| Manes.11G069200 |
| Manes.11G065300 |
| Manes.11G036800 |
| Manes.11G036600 |
| Manes.11G034000 |
| Manes.11G028700 |
| Manes.11G022600 |
| Manes.11G019000 |
| Manes.11G016500 |
| Manes.11G008600 |
| Manes.11G006000 |
| Manes.11G002900 |
| Manes.10G140700 |
| Manes.10G127900 |
| Manes.10G119900 |
| Manes.10G097500 |
| Manes.10G076500 |
| Manes.10G074700 |
| Manes.10G069200 |
| Manes.10G063900 |
| Manes.10G039800 |
| Manes.10G038800 |
| Manes.10G019100 |
| Manes.10G016800 |
| Manes.09G172100 |
| Manes.09G158100 |
| Manes.09G135400 |
| Manes.09G131300 |
| Manes.09G129500 |
| Manes.09G127400 |
| Manes.09G117200 |
| Manes.09G112700 |
| Manes.09G107100 |
| Manes.09G105200 |
| Manes.09G068300 |
| Manes.09G066300 |
| Manes.09G061900 |
| Manes.09G059200 |
| Manes.09G051800 |
| Manes.09G040400 |
| Manes.09G029900 |
| Manes.09G006500 |
| Manes.09G004500 |
| Manes.09G003400 |
| Manes.08G174000 |
| Manes.08G161100 |
| Manes.08G160300 |
| Manes.08G150600 |
| Manes.08G149000 |
| Manes.08G135600 |
| Manes.08G116100 |
| Manes.08G100000 |
| Manes.08G097800 |
| Manes.08G097700 |
| Manes.08G092200 |
| Manes.08G092000 |
| Manes.08G062600 |
| Manes.08G061700 |
| Manes.08G061500 |
| Manes.08G035200 |
| Manes.08G030400 |
| Manes.08G015500 |
| Manes.08G010000 |
| Manes.08G003100 |
| Manes.07G143600 |
| Manes.07G134800 |
| Manes.07G118800 |
| Manes.07G116800 |
| Manes.07G116700 |
| Manes.07G109100 |
| Manes.07G105100 |
| Manes.07G088000 |
| Manes.07G087700 |
| Manes.07G064000 |
| Manes.07G049900 |
| Manes.07G028700 |
| Manes.07G022900 |
| Manes.07G007100 |
| Manes.06G179100 |
| Manes.06G151400 |
| Manes.06G151200 |
| Manes.06G151000 |
| Manes.06G150100 |
| Manes.06G143300 |
| Manes.06G138200 |
| Manes.06G118300 |
| Manes.06G112100 |
| Manes.06G106300 |
| Manes.06G102200 |
| Manes.06G099800 |
| Manes.06G096000 |
| Manes.06G094000 |
| Manes.06G055500 |
| Manes.06G035700 |
| Manes.06G035100 |
| Manes.05G206100 |
| Manes.05G193200 |
| Manes.05G186500 |
| Manes.05G170900 |
| Manes.05G155000 |
| Manes.05G106900 |
| Manes.05G087400 |
| Manes.05G086300 |
| Manes.05G084800 |
| Manes.05G080400 |
| Manes.05G079300 |
| Manes.05G076500 |
| Manes.05G060800 |
| Manes.05G059000 |
| Manes.05G057500 |
| Manes.05G052200 |
| Manes.05G020700 |
| Manes.05G017400 |
| Manes.05G009900 |
| Manes.05G002700 |
| Manes.05G002000 |
| Manes.04G163700 |
| Manes.04G148100 |
| Manes.04G138200 |
| Manes.04G137700 |
| Manes.04G134000 |
| Manes.04G131100 |
| Manes.04G127100 |
| Manes.04G112700 |
| Manes.04G109900 |
| Manes.04G109500 |
| Manes.04G106600 |
| Manes.04G104200 |
| Manes.04G097200 |
| Manes.04G066900 |
| Manes.04G064700 |
| Manes.04G061800 |
| Manes.04G055500 |
| Manes.04G043500 |
| Manes.03G197400 |
| Manes.03G195200 |
| Manes.03G189500 |
| Manes.03G186900 |
| Manes.03G136600 |
| Manes.03G133700 |
| Manes.03G127600 |
| Manes.03G127100 |
| Manes.03G122800 |
| Manes.03G106300 |
| Manes.03G083900 |
| Manes.03G071400 |
| Manes.03G053300 |
| Manes.03G039700 |
| Manes.03G005900 |
| Manes.02G223600 |
| Manes.02G213500 |
| Manes.02G198400 |
| Manes.02G194400 |
| Manes.02G187600 |
| Manes.02G186700 |
| Manes.02G186100 |
| Manes.02G181700 |
| Manes.02G180200 |
| Manes.02G179700 |
| Manes.02G169000 |
| Manes.02G166400 |
| Manes.02G165500 |
| Manes.02G164800 |
| Manes.02G138400 |
| Manes.02G137500 |
| Manes.02G136200 |
| Manes.02G120000 |
| Manes.02G118200 |
| Manes.02G118100 |
| Manes.02G118000 |
| Manes.02G117200 |
| Manes.02G113300 |
| Manes.02G102400 |
| Manes.02G091000 |
| Manes.02G089900 |
| Manes.02G082700 |
| Manes.02G074000 |
| Manes.02G067100 |
| Manes.02G059000 |
| Manes.02G057800 |
| Manes.02G056600 |
| Manes.02G051000 |
| Manes.02G028100 |
| Manes.02G026400 |
| Manes.02G018700 |
| Manes.02G012500 |
| Manes.02G009600 |
| Manes.01G274200 |
| Manes.01G273000 |
| Manes.01G259600 |
| Manes.01G253500 |
| Manes.01G252400 |
| Manes.01G229400 |
| Manes.01G229100 |
| Manes.01G228300 |
| Manes.01G222800 |
| Manes.01G221200 |
| Manes.01G208800 |
| Manes.01G196200 |
| Manes.01G172100 |
| Manes.01G164100 |
| Manes.01G160400 |
| Manes.01G145600 |
| Manes.01G143100 |
| Manes.01G139600 |
| Manes.01G133100 |
| Manes.01G131300 |
| Manes.01G122400 |
| Manes.01G109600 |
| Manes.01G097700 |
| Manes.01G097500 |
| Manes.01G088500 |
| Manes.01G082700 |
| Manes.01G080800 |
| Manes.01G071400 |
| Manes.01G065200 |
| Manes.01G058700 |
| Manes.01G058000 |
| Manes.01G052200 |
| Manes.01G051600 |
| Manes.01G028200 |
| Manes.01G027400 |
| Manes.01G026900 |
| Manes.03G005100 |
| Manes.06G029500 |

## Table S6. Target genes in different modules in WGCNA

| **Gene** | **Module** |
| --- | --- |
| Manes.S108600 | blue |
| Manes.18G041700 | blue |
| Manes.18G036100 | blue |
| Manes.17G025700 | blue |
| Manes.16G131900 | blue |
| Manes.16G050400 | blue |
| Manes.16G040500 | blue |
| Manes.16G030600 | blue |
| Manes.16G003100 | blue |
| Manes.15G156600 | blue |
| Manes.15G151200 | blue |
| Manes.15G116800 | blue |
| Manes.15G076500 | blue |
| Manes.15G050800 | blue |
| Manes.15G034300 | blue |
| Manes.15G011500 | blue |
| Manes.14G150400 | blue |
| Manes.14G078500 | blue |
| Manes.14G064800 | blue |
| Manes.13G132200 | blue |
| Manes.13G052000 | blue |
| Manes.13G019600 | blue |
| Manes.12G131000 | blue |
| Manes.12G023300 | blue |
| Manes.12G015500 | blue |
| Manes.11G162400 | blue |
| Manes.11G065300 | blue |
| Manes.11G008600 | blue |
| Manes.10G039800 | blue |
| Manes.09G172100 | blue |
| Manes.09G105200 | blue |
| Manes.09G029900 | blue |
| Manes.09G003400 | blue |
| Manes.08G100000 | blue |
| Manes.08G097700 | blue |
| Manes.08G092200 | blue |
| Manes.08G092000 | blue |
| Manes.08G030400 | blue |
| Manes.08G010000 | blue |
| Manes.07G134800 | blue |
| Manes.07G088000 | blue |
| Manes.07G064000 | blue |
| Manes.06G151200 | blue |
| Manes.05G193200 | blue |
| Manes.05G087400 | blue |
| Manes.05G059000 | blue |
| Manes.05G017400 | blue |
| Manes.03G071400 | blue |
| Manes.03G039700 | blue |
| Manes.02G198400 | blue |
| Manes.02G186700 | blue |
| Manes.02G166400 | blue |
| Manes.02G138400 | blue |
| Manes.02G118200 | blue |
| Manes.02G118000 | blue |
| Manes.02G117200 | blue |
| Manes.02G082700 | blue |
| Manes.02G067100 | blue |
| Manes.02G057800 | blue |
| Manes.02G056600 | blue |
| Manes.02G051000 | blue |
| Manes.02G026400 | blue |
| Manes.01G273000 | blue |
| Manes.01G222800 | blue |
| Manes.01G221200 | blue |
| Manes.01G196200 | blue |
| Manes.01G097700 | blue |
| Manes.S052900 | brown |
| Manes.18G121700 | brown |
| Manes.18G044500 | brown |
| Manes.17G038000 | brown |
| Manes.16G120400 | brown |
| Manes.16G115300 | brown |
| Manes.16G110200 | brown |
| Manes.16G087600 | brown |
| Manes.15G090500 | brown |
| Manes.14G135100 | brown |
| Manes.13G087000 | brown |
| Manes.12G139200 | brown |
| Manes.12G101400 | brown |
| Manes.12G035600 | brown |
| Manes.10G076500 | brown |
| Manes.09G107100 | brown |
| Manes.09G061900 | brown |
| Manes.09G059200 | brown |
| Manes.09G006500 | brown |
| Manes.08G161100 | brown |
| Manes.08G150600 | brown |
| Manes.08G061700 | brown |
| Manes.07G143600 | brown |
| Manes.07G116700 | brown |
| Manes.07G109100 | brown |
| Manes.07G049900 | brown |
| Manes.06G138200 | brown |
| Manes.06G102200 | brown |
| Manes.06G099800 | brown |
| Manes.06G035700 | brown |
| Manes.05G186500 | brown |
| Manes.04G109500 | brown |
| Manes.04G043500 | brown |
| Manes.03G127600 | brown |
| Manes.03G127100 | brown |
| Manes.01G274200 | brown |
| Manes.01G026900 | brown |
| Manes.S078700 | turquoise |
| Manes.S022900 | turquoise |
| Manes.S012600 | turquoise |
| Manes.18G072700 | turquoise |
| Manes.18G064400 | turquoise |
| Manes.18G057000 | turquoise |
| Manes.18G029700 | turquoise |
| Manes.18G012200 | turquoise |
| Manes.18G002900 | turquoise |
| Manes.17G119500 | turquoise |
| Manes.17G102800 | turquoise |
| Manes.17G100500 | turquoise |
| Manes.17G090500 | turquoise |
| Manes.17G074400 | turquoise |
| Manes.17G065500 | turquoise |
| Manes.17G026600 | turquoise |
| Manes.17G001600 | turquoise |
| Manes.16G129800 | turquoise |
| Manes.16G116200 | turquoise |
| Manes.16G102700 | turquoise |
| Manes.16G074300 | turquoise |
| Manes.16G062800 | turquoise |
| Manes.16G059200 | turquoise |
| Manes.16G042400 | turquoise |
| Manes.16G012700 | turquoise |
| Manes.16G004100 | turquoise |
| Manes.15G185800 | turquoise |
| Manes.15G142500 | turquoise |
| Manes.15G140700 | turquoise |
| Manes.15G132600 | turquoise |
| Manes.15G131100 | turquoise |
| Manes.15G117700 | turquoise |
| Manes.15G101800 | turquoise |
| Manes.15G084200 | turquoise |
| Manes.15G048300 | turquoise |
| Manes.15G039300 | turquoise |
| Manes.15G007400 | turquoise |
| Manes.14G171400 | turquoise |
| Manes.14G143400 | turquoise |
| Manes.14G121400 | turquoise |
| Manes.14G113500 | turquoise |
| Manes.14G063800 | turquoise |
| Manes.14G035500 | turquoise |
| Manes.14G021300 | turquoise |
| Manes.14G019800 | turquoise |
| Manes.14G018200 | turquoise |
| Manes.14G009100 | turquoise |
| Manes.14G006900 | turquoise |
| Manes.13G143900 | turquoise |
| Manes.13G143500 | turquoise |
| Manes.13G138100 | turquoise |
| Manes.13G116500 | turquoise |
| Manes.13G113000 | turquoise |
| Manes.13G084100 | turquoise |
| Manes.13G060800 | turquoise |
| Manes.13G042800 | turquoise |
| Manes.13G010400 | turquoise |
| Manes.13G001300 | turquoise |
| Manes.13G000300 | turquoise |
| Manes.12G134900 | turquoise |
| Manes.12G114400 | turquoise |
| Manes.12G111400 | turquoise |
| Manes.12G110600 | turquoise |
| Manes.12G068900 | turquoise |
| Manes.12G047600 | turquoise |
| Manes.12G043800 | turquoise |
| Manes.12G043700 | turquoise |
| Manes.12G023600 | turquoise |
| Manes.12G009700 | turquoise |
| Manes.11G162900 | turquoise |
| Manes.11G131500 | turquoise |
| Manes.11G096200 | turquoise |
| Manes.11G085500 | turquoise |
| Manes.11G069200 | turquoise |
| Manes.11G036800 | turquoise |
| Manes.11G036600 | turquoise |
| Manes.11G034000 | turquoise |
| Manes.11G028700 | turquoise |
| Manes.11G022600 | turquoise |
| Manes.11G019000 | turquoise |
| Manes.11G016500 | turquoise |
| Manes.11G006000 | turquoise |
| Manes.11G002900 | turquoise |
| Manes.10G140700 | turquoise |
| Manes.10G127900 | turquoise |
| Manes.10G119900 | turquoise |
| Manes.10G097500 | turquoise |
| Manes.10G074700 | turquoise |
| Manes.10G069200 | turquoise |
| Manes.10G063900 | turquoise |
| Manes.10G038800 | turquoise |
| Manes.10G019100 | turquoise |
| Manes.10G016800 | turquoise |
| Manes.09G158100 | turquoise |
| Manes.09G131300 | turquoise |
| Manes.09G129500 | turquoise |
| Manes.09G127400 | turquoise |
| Manes.09G117200 | turquoise |
| Manes.09G068300 | turquoise |
| Manes.09G066300 | turquoise |
| Manes.09G051800 | turquoise |
| Manes.09G040400 | turquoise |
| Manes.09G004500 | turquoise |
| Manes.08G174000 | turquoise |
| Manes.08G160300 | turquoise |
| Manes.08G149000 | turquoise |
| Manes.08G135600 | turquoise |
| Manes.08G116100 | turquoise |
| Manes.08G062600 | turquoise |
| Manes.08G061500 | turquoise |
| Manes.08G015500 | turquoise |
| Manes.08G003100 | turquoise |
| Manes.07G118800 | turquoise |
| Manes.07G116800 | turquoise |
| Manes.07G105100 | turquoise |
| Manes.07G087700 | turquoise |
| Manes.07G028700 | turquoise |
| Manes.07G022900 | turquoise |
| Manes.07G007100 | turquoise |
| Manes.06G179100 | turquoise |
| Manes.06G151400 | turquoise |
| Manes.06G151000 | turquoise |
| Manes.06G150100 | turquoise |
| Manes.06G143300 | turquoise |
| Manes.06G118300 | turquoise |
| Manes.06G112100 | turquoise |
| Manes.06G106300 | turquoise |
| Manes.06G096000 | turquoise |
| Manes.06G094000 | turquoise |
| Manes.06G055500 | turquoise |
| Manes.06G035100 | turquoise |
| Manes.05G206100 | turquoise |
| Manes.05G170900 | turquoise |
| Manes.05G155000 | turquoise |
| Manes.05G086300 | turquoise |
| Manes.05G084800 | turquoise |
| Manes.05G080400 | turquoise |
| Manes.05G079300 | turquoise |
| Manes.05G076500 | turquoise |
| Manes.05G060800 | turquoise |
| Manes.05G057500 | turquoise |
| Manes.05G052200 | turquoise |
| Manes.05G020700 | turquoise |
| Manes.05G009900 | turquoise |
| Manes.05G002700 | turquoise |
| Manes.05G002000 | turquoise |
| Manes.04G163700 | turquoise |
| Manes.04G148100 | turquoise |
| Manes.04G138200 | turquoise |
| Manes.04G137700 | turquoise |
| Manes.04G134000 | turquoise |
| Manes.04G127100 | turquoise |
| Manes.04G112700 | turquoise |
| Manes.04G109900 | turquoise |
| Manes.04G104200 | turquoise |
| Manes.04G097200 | turquoise |
| Manes.04G066900 | turquoise |
| Manes.04G061800 | turquoise |
| Manes.04G055500 | turquoise |
| Manes.03G197400 | turquoise |
| Manes.03G195200 | turquoise |
| Manes.03G189500 | turquoise |
| Manes.03G186900 | turquoise |
| Manes.03G136600 | turquoise |
| Manes.03G133700 | turquoise |
| Manes.03G122800 | turquoise |
| Manes.03G083900 | turquoise |
| Manes.03G053300 | turquoise |
| Manes.03G005900 | turquoise |
| Manes.02G223600 | turquoise |
| Manes.02G213500 | turquoise |
| Manes.02G194400 | turquoise |
| Manes.02G187600 | turquoise |
| Manes.02G186100 | turquoise |
| Manes.02G181700 | turquoise |
| Manes.02G169000 | turquoise |
| Manes.02G165500 | turquoise |
| Manes.02G164800 | turquoise |
| Manes.02G137500 | turquoise |
| Manes.02G136200 | turquoise |
| Manes.02G120000 | turquoise |
| Manes.02G118100 | turquoise |
| Manes.02G113300 | turquoise |
| Manes.02G102400 | turquoise |
| Manes.02G091000 | turquoise |
| Manes.02G089900 | turquoise |
| Manes.02G074000 | turquoise |
| Manes.02G059000 | turquoise |
| Manes.02G028100 | turquoise |
| Manes.02G018700 | turquoise |
| Manes.02G012500 | turquoise |
| Manes.02G009600 | turquoise |
| Manes.01G253500 | turquoise |
| Manes.01G229400 | turquoise |
| Manes.01G229100 | turquoise |
| Manes.01G228300 | turquoise |
| Manes.01G208800 | turquoise |
| Manes.01G172100 | turquoise |
| Manes.01G164100 | turquoise |
| Manes.01G160400 | turquoise |
| Manes.01G145600 | turquoise |
| Manes.01G143100 | turquoise |
| Manes.01G139600 | turquoise |
| Manes.01G133100 | turquoise |
| Manes.01G131300 | turquoise |
| Manes.01G122400 | turquoise |
| Manes.01G097500 | turquoise |
| Manes.01G088500 | turquoise |
| Manes.01G082700 | turquoise |
| Manes.01G080800 | turquoise |
| Manes.01G071400 | turquoise |
| Manes.01G065200 | turquoise |
| Manes.01G058700 | turquoise |
| Manes.01G052200 | turquoise |
| Manes.01G051600 | turquoise |
| Manes.01G028200 | turquoise |
| Manes.01G027400 | turquoise |

**Table S7: Accession numbers of the cultivars RNA-Seq data**

|  |  |
| --- | --- |
| Sample name | Accession number |
| Arg7_D12 | SRR2388947 |
| Arg7_CK12 | SRR2388956 |
| Arg7_CK12_root_ | SRR2495949 |
| Arg7_D12_root | SRR2495950 |
| W14_D12 | SRR2404199 |
| W14_CK12 | SRR2404206 |
| W14_D12_root | SRR2496093 |
| W14_CK12_root_ | SRR2496326 |


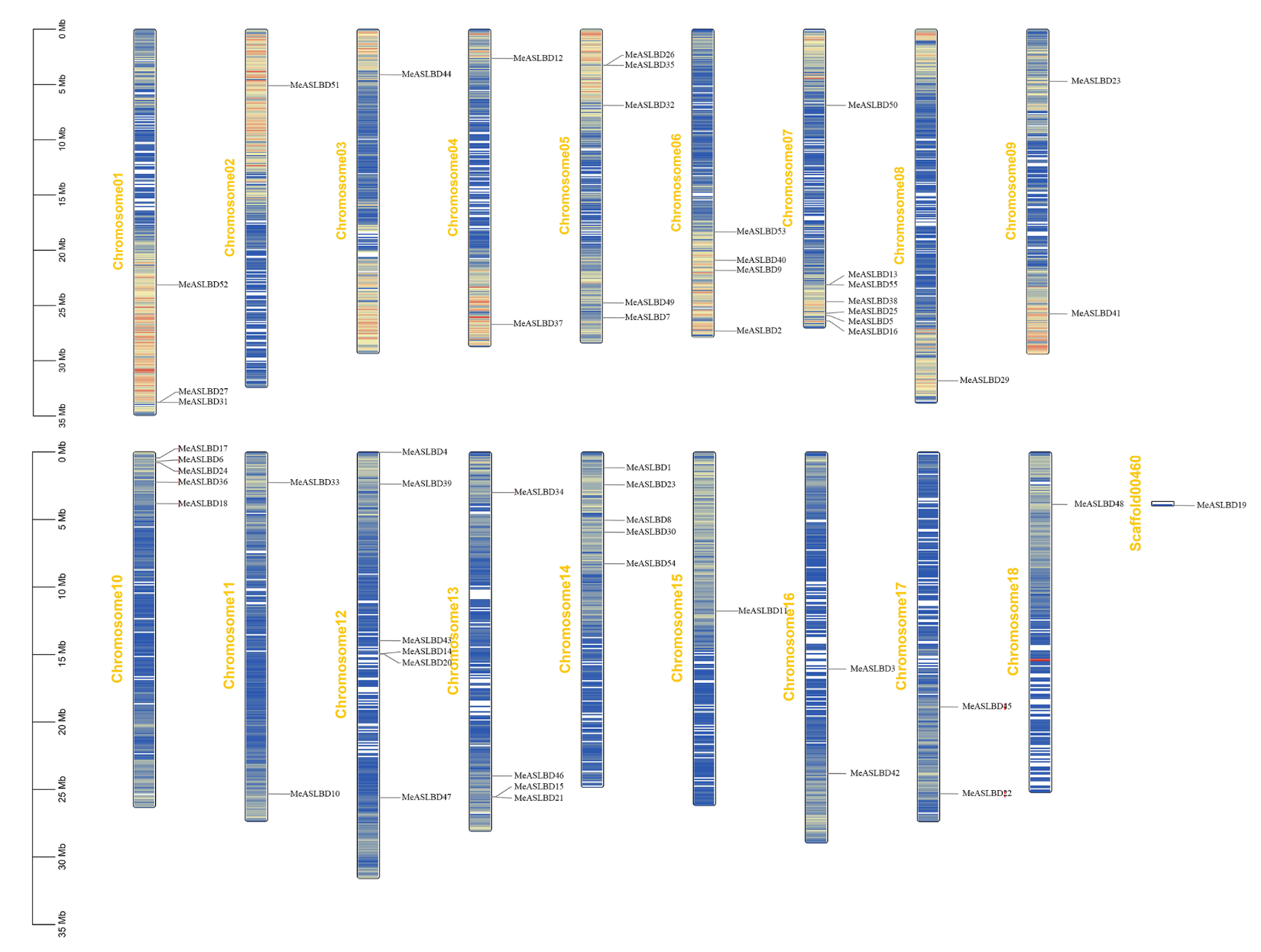


## Fig S1. Localisation of MeLBDs gene family members on cassava chromosomes





## Fig S2. Relative expression of gene LRR in cassava
