# Supplementary material for: Genome-wide analyses of LATERAL ORGAN BOUNDARIES in cassava reveal the role of LBD47 in defence against bacterial blight
Source: PLoS One. 2023 Apr 20;18(4):e0282100. doi: 10.1371/journal.pone.0282100 (PMC10118128; doi:10.1371/journal.pone.0282100)
Supplement: Attachment — Submitted filename: Responses+Reviewers+16012023.pdf [file pone.0282100.s004.pdf]

Response to reviewers

Review Comment to the Author

Reviewer #1: The authors have corrected almost all. However, there are still ìnsuffícient part,
All AtLBD should be written AtASL/LBD, for example,
AtAS2/LBD6, AtASL1/LBD36, AtASL2/LBD10, AtASL3/LBD25, AtASL4/LOB, AtASL5/LBD12, AtASL6/LBD4, AtASL7/LBD11, AtASL8/LBD1, AtASL9/LBD3, AtASL10/LBD13, AtASL11/LBD15, AtASL12/LBD21, AtASL13/LBD24, AtASL14/LBD23, AtASL15/LBD17, AtASL16/LBD29, and so on.
See figure 4 and 5 in Machida et al., 2022 (https://link.springer.com/article/10.1007/s10265-021-01349-6).
Nomenclature with ASLs is important. This is because nomenclature with ASLs is based on the amino acid sequence homology of the AS2/LOB domain. Similar amino acid sequences have been shown to be functionally similar. Also in cassava, it will be possible to infer their functions by comparing with ASLs of Arabidopsis thaliana.
Two types of nomenclature (ASLs and LBDs in Arabidopsis thaliana) should be listed side by side and such nomenclature with ASLs should be advantageous for discussing relationships between the phylogeny and developmental functions of members in the AS2/LOB protein family. For example, at least four known proteins involved in auxin-induced lateral root formation are named ASL18/LBD16, ASL16/LBD29, ASL20/LBD18, and ASL24/LBD33; ASL15/LBD17 is also proposed to play a similar role. All of them are located in the close clades of sub-class Class Ia of A. thaliana in the phylogenetic tree. During pollen development, ASL1/LBD36, ASL2/LBD10, and ASL3/LBD25, all of which belong to the close narrow clades of the tree, control asymmetric cell division during pollen development. Interestingly, another set of ASL members (SCP/ASL29/LBD27 and ASL30/LBD22 in a small clade) are also involved in the same single process, but at different steps during the progression of pollen development. Note that ASL1/LBD36, ASL2/LBD10, and ASL3/LBD25 belong to Class Ia of A. thaliana, and that SCP/ASL29/LBD27 and ASL30/LBD22 belong to Class Ib of A. thaliana. Use of the ASL nomenclature might provide an edge to discussion of evolutionary developmental biology (evo-devo) of the AS2/LOB family.

Response: Thanks for this great point. The two terminology types were listed side by side (ASLs and LBDs). Please see figure 1

Reviewer #3: The manuscript (PONE-D-22-03287) is well-prepared and quite conclusive. The language used in the manuscript is appropriate. The observations have been presented in a proper scientific manner and have been discussed well. Still, there is a scope to uplift the MS, I have a couple of suggestive comments which should further improve the manuscript.

Response: Thanks for taking the time to read our manuscript and for all the constructive comments.

Major comments
[1] The leaf lesion experiment in the VIGS lines is an important experiment, and

currently, the images of the leaves provided are not very convincing. I encourage the authors to provide an image captured under similar light conditions (currently, the brightness and contrast of all the leaf areas are different). Also, Mark the inoculation point with a marker so that the inoculation site and lesion will be visible. Provide a picture of the entire leaf rather than a small section, then zoom in on the necrotic lesion area.

Response: We apologize for the light conditions of the pictures. We have improved the picture with a scale bar, and hopefully, it is convincing enough. Please see picture 12

[2] Line#367 is there any information about these cultivators' abiotic and biotic stress sensitivity/tolerance? If yes, then provide that will strengthen this motive of the experiment.

Response: Thanks for these suggestions. Indeed, it could improve our manuscript. However, we haven't conducted these analyses. And we apologize that we couldn't conduct these analyses due to the time given.

Minor comments

[1] The discussion does not contain the figure references while mentioning the result, and please mention the figure number in the discussion too.

Response: Thanks for pointing these out. We have now referenced the figures in the revised manuscript. Please see the discussion section.

[2] Mention the statistical test performed while analyzing the data to test the significance in Figures 5 and 12

Response: We have now mentioned the statistical test performed. Please see figures 5 and 12

[3] Figure 6- Provide an explanation in the legend about the color code provided in the figure.

Response: We have now added an explanation about the color bar. Please see figure 6 legend

[4] Figure7- Specify tissue type and the variety of cassava used for transcription analysis as mentioned in the figure

Response: We Have now specified the tissue type and cassava varieties. Please see the figure 7 legend.

[5] Figure 8- explain why the MeASLBD19 expression profile is missing (indicated by the gray color) in the disease group.

Response: When we downloaded the expression profile data of cassava LBDs in diseases, we found that the expression abundance of MeASLBD19 was 0, therefore, it was displayed in gray.

[6] Figure 9- The graph seems confusing; in MeASLBD12 and a few others, the relative expression value is significantly high at six h, but it still shows ns. This needs correction. Mention the statistical test performed.

Response: Thanks for pointing out these typos. It has now been revised. Please see figure 9

[7] Fig 12b – provide scale bar.

Response: Thanks; we have provided a scale bar. Please see figure 12b

Line#42 Gene expression is not a good keyword. Replace it.

Response: Thanks. Gene expression has been replaced with expression profiles. Please see line 41

Line#55, "development of fat symmetric," is this fat or flat?

Response: Thanks for pointing out this typo. It has been corrected. Please see line 54

Line#93 what is the basis of dividing into two subclasses? Class II is also divided into subclasses (a-g) which need to be mentioned.

Response: The tree was divided into two classes based on the clades form and previous studies that divided the arabidopsis LOD proteins into two classes: https://academic.oup.com/plphys/article/129/2/747/6110259#265176879

However, the subclasses (a-g) could not be mentioned because they resulted from the current studies.

Line#151. mentions other cassava cultivars, Arg7, W14, and KU50, that were used for gene analysis.

Response: Thanks for pointing it out. We have mentioned the missed cultivars. Please see 150

Line#155 Please specify how many leaves were inoculated per plant.

Response: It has been mentioned. Please see line 154

Line#156- Write bacterial dilution correctly using the superscript.

Response: Thanks for pointing it out. It has been corrected. Please see lines 155

Line#156-Keep consistency in writing optical density here, it is OD600, while in line#194, it is written as (OD600).

Response: We apologised for this inconsistency. We have corrected it with OD_{600} throughout the revised manuscript.

Line#157- replace "hole" with "ring"

Response: Thanks. We have replaced hole with ring. Please see line 156

Line#162 - why only two similar replicates were considered?

Response: We meant, "The experiments were conducted at least twice with identical results".

Line#190-192 this explanation is not required, abridged.

Response: We have abridged the sentence. Please see line 138

Line#235 -Provide the hyperlink for the source code and web page for MCscanX.

Response: Thanks. The source code and web page have been provided. Please see line 235

Line#238 -Provide the hyperlink of the source for the Circos software

Response: Thanks. The hyperlink of the source for the circus software has been provided. Please see line 238

Line#256 provide the source website for the HISAT2 software.

Response: Thanks. The source website for the HISAT2 software has been provided. Please see line 257

Line#378 - RE-frame this sentence as "These results suggest the genotype-dependent tissue expression of these genes." Authors are using genotype and varieties as synonyms which is not the case. For example, in Line#382," cassava cultivars (Arg7 and W14)", these are referred to as Cultivars. However, KU50 and Arg7 are cultivated varieties and W14 wild ancestors. Correct this throughout the MS.

Response: Thanks for pointing these out, and we apologise for the misleading. We have reframed the sentence. Please see lines 378-379

Besides, we have corrected these misleading sentences throughout the revised manuscript.

Line #382-Why the Variety KU50 not included in the expression analysis under drought treatment studies?

Response: Because the expression profiling data (downloaded from NCBI) did not contain the expression pattern of KU50 under drought.

Line #385 "had significant differential expression under drought stress." Mention the fold change in expression compared to the control group.

Response: Thanks for your great comment. Since the folds were not mentioned on the heat map, we think it might be better not to stay consistent with figure 8.

Line# 427- "The 347 genes could be divided into four modules via WGCNA" explain the basis of this division into four modules indicated by different colors.

Response: Highly interconnected gene sets were obtained by WGNCA analysis, and these gene sets were designated as "modules". A total of 4 modules were obtained. Among them, the gray module was considered a non-coexpressed gene set by default; therefore, it was not considered in the analysis. Please see lines 428-429

Reviewer #4: I found the paper to be well-prepared, with excellent and careful data selection, and it addressed all of the previous reviewers' comments with evidence. This MS could be useful for learning more about how ASYMMETRIC LEAVES2-LIKE/LATERAL ORGAN BOUNDARIES (ASL/LBD) transcription factors (TFs) in plants regulates biotic and abiotic stress responses. Therefore, this MS will timely provide important insight to understand ASL/LBD genes in plants under biotic and abiotic stress responses. However, main concerns associated with the MS are as follows:
(I)The authors have corrected most of the things suggested by previous reviewers: 1 and 2. However, I suggest to author please double check the AtLBD. It should be written AtASL/LBD in whole MS including figures and figure legends as also suggested by previous reviewers (twice).

Response: Thanks for taking the time to read our manuscript and for all your suggestions. The two terminology types were listed side by side (ASLs and LBDs) throughout the revised manuscript.

(II)The figures and texts of Figure 1, Figure 2, Figure 3, Figure 10, and Figure S1 are unclear. I suggest authors should include high-resolution figures in their work to make it more understandable to a wider audience.

Response: We apologise for the unclear pictures. We have replaced them with high-resolution throughout the revised manuscript.

Reviewer #5: Mao and Abdoulaye et al - Genome-wide characterization of cassava LATERAL ORGAN BOUNDARIES domain genes revealed LBD47 involved in bacterial blight defense – PLOS ONE

The paper builds the study about genome-wide characterization of LBD genes using HMM profiles while establishing the role of MeLBD47 in plant phytohormone signalling

in cassava. It showed significantly mitigated virulence of cassava bacterial blight (Xam CHN11) through Virus-induced gene silencing (VIGS).

General comment: The manuscript has the topic on the whole well covered and contains interesting set of experiments including VIGS and bioinformatic analyses that can be suitable for publication. Here are my few concerns:

Title: Please reframe the title of the paper. It does not sound very conclusive. One suggestion is "Genome-wide analyses of LATERAL ORGAN BOUNDARIES in cassava reveal the role of LBD47 in defense against bacterial blight".

Response: Thanks for your comments and time to read our manuscript. The title has been reframed with the suggested title. Nevertheless, we would like to mention that most of the comments ref (Lines) were based on the first revised manuscript. Therefore we have already revised some parts previously.

Line 23: 'T' small case in Arabidopsis thaliana

Response: Thanks for pointing out this typo. It has been corrected. Please see line 21

Line 32: 'MeLBD47 was selected…'. How was it selected? Randomly? If yes, please mention that and if no, mention what was the basis of its selection for functional analyses?

Response: MeLBD47 was selected based on the expression level under disease treatment and drought stress.

Line 32 and 33: MeLBD47 is italicised somewhere and somewhere not. Please maintain uniformity.

Response: We apologise for the inconsistency. We have revised it throughout the revised manuscript.

Line 65: Add space between esculentaCrantz

Response: It has been corrected

Line 72: 'extand' misspelled

Response: It has been corrected

Line 99: $2^{-\Delta CT}$ or $2^{-\Delta\Delta CT}$?

Response: It has been corrected

Line 197-201: Information is repeated from Introduction.

Response: It has been revised.

Line 283: ‘leaf’, not ‘leave’

Response: Thanks for pointing out these typos. It has been corrected throughout the revised manuscript.

Line 343: ‘inoculated with’, not ‘Inoculated’

Response: Thanks for pointing out these typos. It has been corrected throughout the revised manuscript.

Discussion: Authors have mentioned in the introduction that this information will provide invaluable insights for further studies. I would suggest that the paper will be more informative if few lines are added in the discussion part about how this information can be put in use and what kinds of further studies can be done.

Response: Thanks for these great suggestions. We have revised the discussion section accordingly, and hopefully, it meets the requirements.
