# Supplementary material for: Genome-wide analyses of LATERAL ORGAN BOUNDARIES in cassava reveal the role of LBD47 in defence against bacterial blight
Source: PLoS One. 2023 Apr 20;18(4):e0282100. doi: 10.1371/journal.pone.0282100 (PMC10118128; doi:10.1371/journal.pone.0282100)
Supplement: Attachment — Submitted filename: Response to review.docx [file pone.0282100.s002.docx]

**Reviewers' comments:**

**Reviewer's Responses to Questions**

**Comments to the Author**

Reviewer #1: The authors reported the AS2/LOB gene family in cassava (Manihot esculenta), in which members contain plant specific AS2/LOB domain. They identified and characterized 55 ASL/LBD genes (MeLBD1 to MeLBD55) from cassava genome using hidden Markov model profiles. They showed that the expression profiles of these genes were highly diverse. Weighted gene co-expression network analysis (WGCNA) of target genes and promoter analysis suggest that some members of cassava MeLBDs may be involved in hormone and stress responses. Furthermore, they described that the promoter analysis suggested that cassava MeLBDs may be involved in the plant phytohormone signal response.

Major points:

**Response:** We thank the reviewer for the time and effort to read our manuscript and for the constructive comments.

(1) Genome-wide characterisation of the AS2/LOB gene family should be very useful for understanding the function of these genes in cassava. To refine these analysis, they should refer to four papers as following on this subject.

Bowman et al., 2017. Insights into Land Plant Evolution Garnered from the Marchantia polymorpha Genome. Cell 171, 287-304 doi.org/10.1016/j.cell.2017.09.030

Machida Y., Suzuki T., Sasabe M., Iwakawa H., Kojima S., Machida C. Arabidopsis ASYMMETRIC LEAVES2 (AS2): roles in plant morphogenesis, cell division, and pathogenesis J. Plant Research 2022 135:3–14 (doi.org/10.1007/s10265-021-01349-6）

Coudert, Y.; Dievart, A.; Droc, G.; Gantet, P. ASL/LBD phylogeny suggests that genetic mechanisms of root initiation downstream of auxin are distinct in lycophytes and euphyllophytes. Mol. Biol. Evol. 2013, 30, 569–572.

Matsumura Y, Iwakawa H, Machida Y, Machida C (2009) Characterization of genes in the ASYMMETRIC LEAVES2/LATERAL ORGAN BOUNDARIES (AS2/LOB) family in Arabidopsis thaliana, and functional and molecular comparisons between AS2 and other family members. Plant J 58:525–537

According to these papers, LATERAL ORGAN BOUNDARIES domain genes should be written ASYMMETRIC LEAVES2-like/LATERAL ORGAN BOUNDARIES DOMAIN (ASL/LBD) genes, MeLBD should be MeASLBD and the LBD gene family should be the AS2/LOB gene family.

**Response:** Fair point. LATERAL ORGAN BOUNDARIES domain genes have been replaced with ASYMMETRIC LEAVES2-like/LATERAL ORGAN BOUNDARIES DOMAIN (ASL/LBD) genes throughout the manuscript. In addition, MeLBD and the LBD gene family were replaced with MeASLBD and AS2/LOB gene family, respectively, throughout the manuscript.

In Phylogenetic trees of figure 1, all AtLBD should be written AtASL/LBD, for example, AtASL1/LBD36, AtASL2/LBD10, AtASL3/LBD25, AtASL4/LOB, AtAS2/LBD6, which are classified into Class Ib in this paper.

**Response:** All AtLBD were replaced with the appropriate abbr. in figure 1 and throughout the manuscript.

If so, it should be very useful to understand the evolutionary relationship between the ASL/LBD proteins of cassava and those of arabidopsis. In addition, it should be useful for the functionary relationship between them, because the authors can recognize that those with similar numbers are functionally close to each other in arabidopsis.

**Response:** Thanks for this sugestions. To make it less speculative, we have discussed some well-studied *Arabidopsis* LBD genes' relationship with cassava. Please see the discussion section.

In figure 1, MeLBD should be MeASLBD, for example, MtASLBD1, MtASLBD2, MtASLBD3 like Marchantia (Bowman et al., 2017).

**Response:** MeLBD is replaced with MeASLBD in figure 1 and throughout the revised manuscript

(2) AS shown in figure 2, it is very interesting that members of the MeASLBD gene family have one to four exons. Class Ia contain one putative splicing site in the AS2/LOB domain at the same site as that in arabidopsis (see Figure 1b in Matsumura et al., 2009) . In arabidopsis a comparison of the sequences of the cloned cDNAs with those of the corresponding genes revealed that 29 genes contained introns in their coding regions, and these genes could be divided into five subtypes on the basis of the positions of the introns in the coding regions (subtypes A–E). The subtype in arabidopsis seems to be corresponded to subtypes in cassaba. They should describe the relationship of exons/introns in their AS2/LOB domain regions between cassaba and arabidopsis.

**Response:** Thanks for this valuable suggestion. We have described the relationship of the splicing sites in the coding regions of *MeASLBD* and *Arabidopsis* AS2/LOB domain. Please see the discussion section and “Evolutionary relationship and gene structure analysis of the MeASLBD genes” section.

(3) As shown in Figure 3, multiple sequence alignment of the MeAS2/LOB domain sequences using DNAMAN showed that all sequences contained three subdomeins named C Block, GAS block and L-rich Block. According to the recent two papers as follows, the authors should change the name of subdomain in the AS2/LOB domain. C Block should be written ZF-motif, GAS block should be ICG/GAS-region, L-rich Block should be LZL-region

Machida et al., (2022) J. Plant Research 135:3–14 (doi.org/10.1007/s10265-021-01349-6）

Chen WF, Wei XB, Rety S, Huang LY, Liu NN, Dou SX, Xi XG (2019) Structural analysis reveals a "molecular calipers" mechanism for a LATERAL ORGAN BOUNDARIES DOMAIN transcription factor protein from wheat. J Biol Chem 294:142–156

**Response:** C Block, GAS block, and L-rich Block have been renamed throughout the manuscript.

(4) The authors described that potential target genes were identified via promoter analysis, and a co-expression network was constructed along with MeLBDs based on a transcriptome dataset derived from pathogen treatment. They found that the MeLBD47 gene expression was highly induced following inoculation with cassava bacterial blight pathogen (XamCHN11), selected for functional analysis. And then the VIGS technique was applied to determine the function of the MeLBD47 gene following XamCHN11 treatment.

They described that one thousand four hundred forty genes contain a 5' GCGGCG 3' motif in the only promoter of the cassava genome, a putative motif bound by ASL/LBD genes. Although some members of the AS2/LOB family have been shown interaction to 5' GCGGCG 3' motif (Husbands et al., 2007; Vial-Pradel et al., 2018; Chen et al., 2018), function of interaction to 5' GCGGCG 3' motif is not known yet. Vial-Pradel et al. found that AS2 binds specifically the sequence containing 5' GCGGCG 3' motif in exon 1 of ETT/ARF3 (direct target of AS2), and that the binding requires the ZF-motif in AS2 that is structurally similar to the zinc finger-CxxC domain in vertebrate DNA methyltransferase1. In addition, Vial-Pradel et al. showed that AS2 did not bind to DNA sequence containing 5' GCGGCG 3' like motif of promoter region of ETT/ARF3.

The authors may examine binding to DNA sequence containing 5' GCGGCG 3' motif in candidate of target of MeASLBD47 and/or discuss about function of binding to DNA sequence containing 5' GCGGCG 3' motif.

Vial-Pradel S., Keta S., Nomoto M., Luo L., Takahashi H., Suzuki M., Yokoyama Y., Sasabe M., Kojima S., Tada Y., Machida Y., Machida C. Arabidopsis zinc-finger-like protein ASYMMETRIC LEAVES2 (AS2) and two nucleolar proteins maintain gene body DNA methylation in the leaf polarity gene ETTIN (ARF3). Plant Cell Physiol. 59(7):1385-1397 (2018).

**Response:** Fair point, and thanks for this valuable point. However, we could not achieve this analysis due to the timing given.

(5) In Figure 11b, the authors showed that the leaves of pCsCMV-LBD47 plants were inoculated with XamCHN11, and pCsCMV-LBD47 showed no obvious lesions after three days, although lesions appeared in the pCsCMV-NC (control). They should show quantitative data.

**Response:** The quantitative data is added. Please see figure 11.

(6) Line 349, The authors wrote "Among them, the expressions of HSFC1 HSP90 and GAD1 were significantly downregulated, indicating that they were affected by the effect of VIGS." However, HSP90 was not downregulated, but upregulated.

**Response:** We apologize for this typo. It has been corrected. Please see lines 533-534

(7) There is no Figure legends. The authors should write Figure legends for Fig 1-11.

**Response:** We apologize for the missing legends. The figure legends have been added. Please see the figures and tables section

(8) In References, The authors did not write name of journal. They should write name of journal of each reference.

**Response:** The journal names have been added. Please see the reference section

(9) Arabidopsis Thaliana should be Arabidopsis thaliana.

**Response:** We apologize for this typo. It has been corrected throughout the manuscript.

**Reviewer #2:** This paper "Genome-wide characterisation of cassava LATERAL ORGAN BOUNDARIES domain genes revealed LBD47 involved in bacterial blight defense" investigates the LBD genes (MeLBD1 to MeLBD55) in cassava genome using hidden Markov model profiles. A lot of papers have been published on cassava plants infected with Xanthomonas axonopodis. The work showed methodological lacks where necessary, unnecessary statements, and weak discussion. The experimental design data has been poorly explored without any statistical analysis and result description. Moreover, the manuscript has a lot of language and editing issues, which should be corrected and needs extensive revision. In some lines, the inappropriate use of English language makes the manuscript really hard to follow.

**Response:** We thank the reviewer for the time and effort to read our manuscript and for the constructive comments. Furthermore, the manuscript language has been edited.

Title:

The inappropriate use of English language makes difficult to follow the title. It should be precise and understandable.

**Response:** We apologize for the misleading. The title has been revised: A genome-wide analysis of the ASYMMETRIC LEAVES2-LIKE/LATERAL ORGAN BOUNDARIES (ASL/LBD) gene family in cassava (Manihot esculenta) and expression profile under biotic and abiotic stress

Abstract:

Abstract is not well written, there is no proper background, methods, directly results are discussed without any conclusion. There are lots of English language issues that makes the abstract really hard to follow. Some very basic scientific writing errors are there, such as on line 23 "Arabidopsis Thaliana" is written. It should be written as Arabidopsis thaliana.

**Response:** Thanks for pointing it out, and the typos have been corrected. In addition, the abstract has been revised accordingly. Please see the abstract.

Keywords:

Authors should use full name instead of abbreviation in this part.

**Response:** Thanks for pointing it out. The full names were used instead of the abbreviation.

Introduction:

The Introduction is poorly written and has many language issues, which need to be corrected. It is advised to get help of English editing service. The literature discussed in one sentence is mostly disconnected from the next sentence, hampering the reading fluency e.g. from Line 43 to 52, 55 to 57, 66 to 67, etc. At some places the meaning of sentence is ambiguous. The information provided need improvement by adding literature relevant to the study conducted. The study objectives are lacking. Authors must clearly present the hypothesis of their study.

**Response:** Thanks for pointing these out. We have improved the redaction following the requirements. Hopefully, it is clear enough. Please see the introduction section

Methods:

The methods description also needs improvements. The authors should use scientific language to write protocols and procedures. More clarity is needed in this section like:

• How the Xanthomonas axonopodis infection was given and at which CFU?

**Response:** Cassava plants were inoculated by dropping 10‐µL of *Xam*CNH11 suspension of 1 × 108 CFU/mL [optical density at 600 nm (OD600) = 0.1] in a 2‐mm‐diameter hole. Please see line 151-153

• How symptoms were monitored post-inoculation and evaluated according to a severity scale?

**Response:** To quantify the lesion area, typical Xam symptoms, such as necrosis and chlorosis around the inoculation point, were considered. At 3 dpi, lesions were measured on eight leaves per treatment and then plotted.

• How the HMM profile was created?

**Response:** The HMM profile was built using the HMMER software (http://hmmer.org/). Please see lines 208-210

• How authors analyzed the conserved domains?

**Response:** The conserved motifs were analyzed using MEME (version 5.4.1). Please see lines 208-210

• Which model authors used to construct the phylogenetic tree?

**Response:** The substitution model used to construct the tree was G + I. Please see lines 228

• What was the optimum width of each motif?

**Response:** The optimum width is 25-50. Please see lines 235

• From where authors retrieve the files for analyzing exon-intron structure?

**Response:** exon-intron date file were retrieved from phytozome (http://www.phytozome.net)

• Authors should express the syntenic relationship of the gene pairs and their respective loci in cassava genome.

**Response:** Collinearity analysis has been inferred. Please see Figure 12

• From where authors retrieve GFF3 file for chromosomal location?

**Response:** GFF3 file were retrieved from Ensembl Plants (http://plants.ensembl.org/index.html). Please see 242

• Microcollinearity is valuable to understand the investigation of gene loss during evolution or the evolution of specific genes in a local region. Therefore, authors must do TGT (Triticeae-Gene Tribe) analysis to trace the origin history of the target gene, and also analyze gene pair.

**Response:** Fair point, and thanks for this valuable point. However, we could not achieve this analysis due to the timing given.

Results:

Result part need substantial improved. The inappropriate use of English language makes the results hard to follow. For example, from lines 178 to 180, etc. Results are ambiguous. The authors must double check the information given in the manuscript as the methodology section saying something different than what is explained in results section including figures.

**Response:** We apologize for the misleading. The results redaction has been improved, and we have double-checked the results throughout the manuscript. Please see the results section

• The information given in Table 1 is already available on "iTAK - Plant Transcription factor & Protein Kinase Identifier and Classifier". What new authors have done?

**Response:** Table 1 has been moved to the supplementary Table (Table S7)

• Figures are not clear. Authors must add high resolution figures to make them understandable. Authors must double check whether the information in the figures are same as they have been written in the results section.

**Response:** We apologize for the low resolution of the pictures provided. High-resolution pictures were added. Besides, we have double-checked the results and figures information.

• There are no figure legends. Authors are advised to add them.

**Response:** Sorry, the missing legends have been added. Please see the Figures and tables section

• Were the LBD genes groups evenly distributed among chromosomes or different homologous groups?

**Response:** The MeASLBD genes were distributed on the 18 cassava chromosomes through an analysis of chromosomal localization.

• Methodology and results of synteny analysis are missing while there is heading in the results.

**Response:** The genomic and GFF3 data of Cassava and Arabidopsis thaliana were Downloaded from Ensembl Plants (http://plants.ensembl.org/index.html). TBtools was used to analyze the synteny. The homologous regions of the MeASLBD and AtLBDs genes were identified with the MCScanX program to determine the level of synteny. Please “Chromosomal location, gene duplication event, gene collinearity, and synteny analysis” section

• Authors must analyze the duplication event

**Response:** Thanks. The duplication event is analysed. Please see the result section

• The results of Exon–intron structure divergences are missing. Authors have only discussed exons but there is no information regarding the introns in all these genes.

**Response:** Exon-introns structures divergences were now included. Please see the discussion section and “Evolutionary relationship and gene structure analysis of the MeASLBD genes” section.

• The authors must correlate the phylogenetic relationship among LBDs with exon–intron structure

**Response:** Thanks for the suggestions. Exon-intron structures were described. Please see **discussion section**

• In methodology, authors wrote that "The cassava cultivar used in this study was South China 8 (SC8)" while in the results the authors wrote "The expression profiles of MeLBDs in different tissues (leaves and roots) of the cassava cultivar Argentina 7 (Arg7), wild subspecies (W14) and variety Kasetsart University 50 (KU50) were analysed". Which information is correct?

**Response:** SC8 cultivar was used in this study. Arg7, W14 and KU50 data were retrieved from NCBI database https://www.ncbi.nlm.nih.gov/geo/query/acc.cgi?acc=GSE93098

• What does the heatmap in Figure 6 shows? There is no methodology for this. Results are different from what is said in the methodology.

**Response:** Figure 6 shows the expression patterns of ASL/LBD under different stresses in cassava. We downloaded these transcriptional data from NCBI's GEO database. Rstudio was used to infer the heatmap.

• The results described in "Expression analysis of MeLBD genes in different tissues and organs" are difficult to understand as the figure which is cited in this don't show the similar information.

**Response:** We apologized for the misleading and we have tried as much as we can to clarify it.

• There is no methodology for drought. How it appear in the results section?

**Response:** Drought treatment data were downloaded from NCBI, this is public data.

• The transcriptome analysis was done with which treatments and on which plants?

**Response:** The transcriptome data were retrieved from the NCBI database https://www.ncbi.nlm.nih.gov/geo/query/acc.cgi?acc=GSE93098

• When silencing of MeLBD47 gene was done? What was its methodology?

**Response:** The silencing method was achieved through VIGS. Please see “Generation of pCsCMV VIGS construct and agroinfiltrationGeneration of pCsCMV VIGS construct and agroinfiltration” section

• How the statistical analysis was performed?

**Response:** We selected three sets of biological replicates and three sets of technical replicates. Statistics analysis was conducted using SPSS software. Please see line 174

• What does the stars on the figure 8 show?

**Response:** Thanks for pointing it out. The asterisk showed a significance level. It has been added in figure 8 legends.

Discussion:

Discussion is very poorly written. This section needs substantial improvement in terms of English language and relevant review of literature. In its current form, it is written much more like introduction and results. In text citation also need revision.

**Response:** The discussion has been revised and improved. Hopefully, it meets the requirements. Please see the discussion section
