# Supplementary material for: Genome-wide analyses of LATERAL ORGAN BOUNDARIES in cassava reveal the role of LBD47 in defence against bacterial blight
Source: PLoS One. 2023 Apr 20;18(4):e0282100. doi: 10.1371/journal.pone.0282100 (PMC10118128; doi:10.1371/journal.pone.0282100)
Supplement: Attachment — Submitted filename: Responses+To+Reviewers+11092022.docx [file pone.0282100.s003.docx]

**Cover Letter**

**Dr. Yinhua Chen**

Hainan Key Laboratory for Sustainable Utilisation of Tropical Bioresources,

College of Tropical Crops,

Hainan University,

Haikou 570228, Hainan, China

**11.09.2022**

**Dear Editor-in-Chief,**

With great pleasure, I re-submit the revised version of the manuscript ID PONE-D-22-03287 entitled "Genome-wide characterisation of cassava LATERAL ORGAN BOUNDARIES domain genes revealed LBD47 involved in bacterial blight defense" for consideration by **PlosOne**. Firstly, I would like to thank you for the opportunity to revise this manuscript. I highly appreciated the constructive comments and fruitful suggestions provided by each reviewer. The manuscript has certainly benefited from these insightful suggestions. Therefore, I look forward to working with you and the reviewers to move this manuscript for publication in the journal of **PlosOne***.*

We have responded to all the reviewer's comments. Major improvements were made accordingly.

Review Comment to the Author

Reviewer #1: The authors have corrected some of the things I pointed out. However, there are still many shortcomings. In addition, there are many inadequate understandings of important papers published in the past.

Although I suggested four papers to be concerned, the authors did not cite the three papers as follows. They should read precisely, cite them and add them in References.

Bowman et al., 2017. Insights into Land Plant Evolution Garnered from the Marchantia polymorpha Genome. Cell 171, 287-304 doi.org/10.1016/j.cell.2017.09.030

Machida Y., Suzuki T., Sasabe M., Iwakawa H., Kojima S., Machida C. Arabidopsis ASYMMETRIC LEAVES2 (AS2): roles in plant morphogenesis, cell division, and pathogenesis. J. Plant Research 2022 135:3&ndash;14 (doi.org/10.1007/s10265-021-01349-6)

Coudert, Y.; Dievart, A.; Droc, G.; Gantet, P. ASL/LBD phylogeny suggests that genetic mechanisms of root initiation downstream of auxin are distinct in lycophytes and euphyllophytes. Mol. Biol. Evol. 2013, 30, 569&ndash;572.

**Response:** Thanks for pointing out these omissions. Indeed, these references outstandingly studied the origin and evolution of the LOB domain. Therefore, these references were cited throughout the revised manuscript.

Although I suggested that all AtLBD should be written AtASL/LBD in Figure 1, the authors wrote only 15 genes out of 43 genes as AtASL/LBD. They should change all AtLBD to AtASL/LBD.

**Response:** Thanks for pointing it out. All AtLBD were changed to AtASL/LBD. Please see figure 1

Although I pointed, the authors did not change them in Figure 3. They should replace. C Block should be written ZF-motif, GAS block should be ICG/GAS-region, L-rich Block should be LZL-region.

**Response:** We apologize for this omission. C Block, GAS block, and L-rich Block were changed into ZF-motif, ICG/GAS-region, and LZL-region, respectively, throughout the manuscript and in figure 3. Please see figure 3.

Although the authors described that they could not achieve this analysis due to the timing given, they should understand precisely and cite the following two papers at least.

Vial-Pradel S., Keta S., Nomoto M., Luo L., Takahashi H., Suzuki M., Yokoyama Y., Sasabe M., Kojima S., Tada Y., Machida Y., Machida C. Arabidopsis zinc-finger-like protein ASYMMETRIC LEAVES2 (AS2) and two nucleolar proteins maintain gene body DNA methylation in the leaf polarity gene ETTIN (ARF3). Plant Cell Physiol. 59(7):1385-1397 (2018).

Chen WF, Wei XB, Rety S, Huang LY, Liu NN, Dou SX, Xi XG (2019) Structural analysis reveals a &quot;molecular calipers&quot; mechanism for a LATERAL ORGAN BOUNDARIES DOMAIN transcription factor protein from wheat. J Biol Chem 294:142&ndash;156

**Response:** Thanks for these suggestions. These references were cited throughout the revised manuscript.

They did not complete to correct yet. They should correct all.

**Response:** We apologize for omitting these important points. Efforts have been made to correct the misinformation. We hope our current revised manuscript meets the publication criteria in PlosOne.
